# The SWI/SNF Subunit INI1 Contains an N-Terminal Winged Helix DNA Binding Domain that Is a Target for Mutations in Schwannomatosis

**DOI:** 10.1016/j.str.2015.04.021

**Published:** 2015-07-07

**Authors:** Mark D. Allen, Stefan M.V. Freund, Giovanna Zinzalla, Mark Bycroft

**Affiliations:** 1 MRC Laboratory of Molecular Biology, Francis Crick Avenue, Cambridge Biomedical Campus, Cambridge CB2 0QH, UK; 2 Centre for Advanced Cancer Therapies, Department of Microbiology, Cell and Tumour Biology and Science for Life Laboratory, Karolinska Institutet, Tomtebodavägen 23, Stockholm 171 65, Sweden

## Abstract

SWI/SNF complexes use the energy of ATP hydrolysis to remodel chromatin. In mammals they play a central role in regulating gene expression during differentiation and proliferation. Mutations in SWI/SNF subunits are among the most frequent gene alterations in cancer. The INI1/hSNF5/SMARCB1 subunit is mutated in both malignant rhabdoid tumor, a highly aggressive childhood cancer, and schwannomatosis, a tumor-predisposing syndrome characterized by mostly benign tumors of the CNS. Here, we show that mutations in INI1 that cause schwannomatosis target a hitherto unidentified N-terminal winged helix DNA binding domain that is also present in the BAF45a/PHF10 subunit of the SWI/SNF complex. The domain is structurally related to the SKI/SNO/DAC domain, which is found in a number of metazoan chromatin-associated proteins.

## Highlights

• INI1 and its metazoan homologs contain a variant winged helix DNA binding domain
• A homologous domain is present in the BAF45a/PHF10 subunit of the SWI/SNF complex
• Structurally related domains are found in other metazoan chromatin-associated proteins
• INI1 mutations that cause schwannomatosis map to the winged helix domain

## Teaser

Allen et al. show that the INI1/hSNF5/SMARCB1 subunit of the human SWI/SNF chromatin-remodeling complex contains a variant winged helix DNA binding domain. Mutations in INI1 that cause the tumor-predisposing syndrome schwannomatosis perturb the fold or disrupt the function of this domain.

## Introduction

Enzymes that use the energy of ATP hydrolysis to remodel chromatin play a central role in regulating eukaryotic gene expression (Narlikar et al., 2013). SWI/SNF chromatin-remodeling complexes were first identified in yeast, but have subsequently been found in all eukaryotic linages. In mammals SWI/SNF-like complexes bind numerous promoters and enhancers, and control the expression of many of the genes that regulate cell growth and differentiation (Tolstorukov et al., 2013). Human SWI/SNF-like complexes consist of invariant core subunits, which are conserved in yeast, as well as a number of tissue-specific subunits, many of which are unique to metazoa (Euskirchen et al., 2012). A wide range of different complexes can be formed, which are generally divided into BAF variants that contain ARID1A or ARID1B, or PBAF variants, which contain ARID2 (BAF200), PBRM1 (BAF180), and BRD7. Changes in subunit composition often accompanies cell differentiation, with particular sets of subunits being required for stem cell self-renewal and proliferation, and others being associated with differentiated post-mitotic tissues. Mutations in SWI/SNF subunits are frequently associated with cancer (Masliah-Planchon et al., 2015). For example, germline alterations (mutations or deletions) of the gene coding for INI1 (also referred to as hSNF5 due to its similarity to the yeast protein SNF5, SMARCB1, or BAF47), one of the core subunits of the complex, predispose individuals to rhabdoid tumors, highly aggressive cancers primarily affecting young children (Versteege et al., 1998). Mutations in the *INI1* gene also cause the tumor suppressor syndrome schwannomatosis, which is characterized by mostly benign tumors of the CNS that typically develop in the second or third decade of life (Hulsebos et al., 2007). Rhabdoid tumors are caused by either deletion of the *INI1* gene, nonsense mutations, or frameshift mutations that introduce a novel stop codon. Mutations within the *INI1* gene that are linked to schwannomatosis, in contrast, are missense mutations and in-frame deletions affecting residues in the N-terminal portion of the protein (Bacci et al., 2010; Christiaans et al., 2011; Hadfield et al., 2008; Smith et al., 2012b, 2014). Several of the mutant proteins have been shown to retain some activity (Smith et al., 2012a).

Despite the importance of INI1, little is known about its function. It is not essential for the assembly of the complex or for its basal remodeling activity. Analysis of its sequence has not revealed any obvious functional domains. It participates in a number of protein-protein interactions involving transcription factors such as c-MYC and GLI1 as well as transcriptional cofactors (Cheng et al., 1999; Jagani et al., 2010). Here, we show that the N-terminal region of INI1, in which the schwannomatosis missense mutations and in-frame deletions are located, contains a hitherto unidentified winged helix DNA binding domain that is also found in metazoan INI1 homologs and the BAF45a subunit of the PBAF complex.

## Results

### INI1 and Its Metazoan Homologs Contain a Novel N-Terminal Domain

Members of the INI1/SNF5 family are all characterized by two imperfect 60-amino-acid repeats followed by a putative coiled coil. The region N-terminal to the repeats is more diverse. On examining the sequence of the N-terminal portion of INI1 we noted a conserved region, only found in metazoan proteins, of approximately 100 amino acids that is predicted to be structured by the program JPRED (Cole et al., 2008). Expression of residues 1–115 of INI1 in *Escherichia coli* produced a monomeric protein that gave well-dispersed nuclear magnetic resonance (NMR) spectra characteristic of a globular protein, confirming that these residues compose an autonomously folded domain. To determine whether this domain is present in other proteins, we undertook a detailed sequence profile search using the PSI-BLAST program (Altschul et al., 1997). Using residues 10–110 of INI1 as the query sequence recovered, in addition to INI1 homologs from animals, a region of BAF45a (also known as PHF10), another subunit of the human SWI/SNF complex (e = 10^−6^, iteration 4). A reciprocal search with this portion of BAF45a retrieved the N-terminal domain of INI1 and metazoan INI1-like proteins. BAF45a is a member of a family of mutually exclusive SWI/SNF subunits (BAF45a, b, c, d), unique to metazoans, all of which contain tandem PHD fingers at their C termini. Complexes contain BAF45a appear to be associated with dividing cells, during neural development; for example, BAF45a is switched for BAF45b or BAF45c as proliferating neural stem and progenitor cells transition into post-mitotic neurons (Lessard et al., 2007). The region of BAF45a similar in sequence to INI1 is N-terminal to the PHD domains and is not found in other family members. It is part of the SAY domain, so named because it was first identified in the *Drosophila* BAF45a homolog SAYP (Shidlovskii et al., 2005).

### The INI1 N-Terminal Domain Has a DHD-like Winged Helix Fold

**Figure 1:**
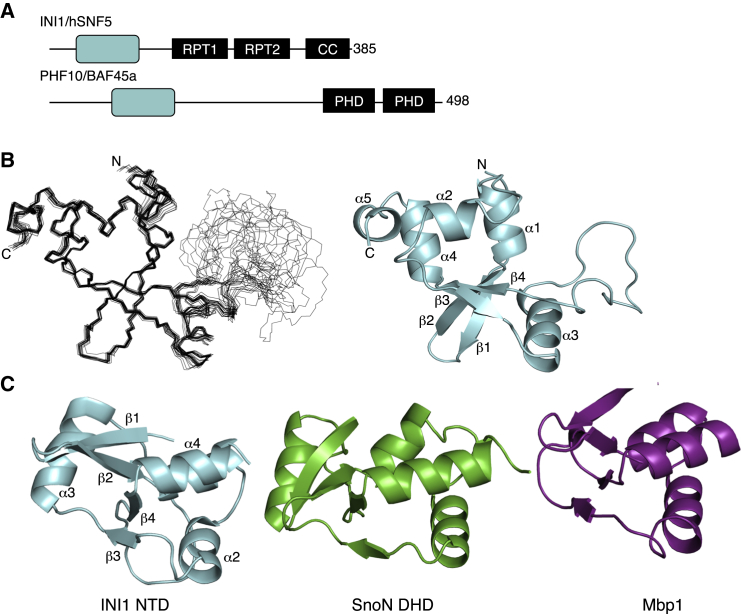
Structure of the N-Terminal Domain of INI1 (A) Schematic representation of the domain organization of INI1 and BAF45a/PHF10. (B) Superposition of the backbone traces of residues 10–110 of the 20 lowest-energy structures of the INI1 N-terminal domain (left) and a ribbon diagram of a representative structure (right). (C) Comparison of the structures of, from left to right, the INI1 N-terminal domain, the DHD domain of SnoN, and Mbp1.

To learn more about this domain, we determined its structure using NMR spectroscopy (Table 1). An overlay of the backbone traces of the 20 lowest-energy structures along with a cartoon representation of a representative structure are shown in Figure 1B. Residues 10–110 of INI1 form a compact ββααβαβαα fold in which the strands are hydrogen bonded in the order β1-β2-β4-β3. Helices 1, 2, 4, and 5 pack onto one side of the sheet and helix 3 packs onto the other side. The structure is well defined except for the loop between helix 3 and strand 4, which ^1^H,^15^N-NOE (nuclear Overhauser effect) measurements show is highly mobile (Figure S1). Comparing the structure with known structures using the program DALI (Holm and Rosenstrom, 2010) revealed significant similarities to the dachshund homology domain (DHD) of SnoN (*Z* score of 6.2). The SnoN domain is a member of a family of structurally related domains found exclusively in metazoan chromatin-associated proteins that includes the DHD domains of DACH1 and the SKI proto-oncogene (Kim et al., 2002; Nyman et al., 2010; Wilson et al., 2004) as well as domains in the *BCL6* co-repressor BCOR (Junco et al., 2013) and DNTTIP1, a subunit of the HDAC1:MIDEAS co-repressor complex (Itoh et al., 2015). The INI1 N-terminal domain also shows significant structural similarity to the winged helix DNA binding domain of the Mbp1 family of yeast cell cycle regulatory proteins (Taylor et al., 1997; Xu et al., 1997). Winged helix domains are found in many DNA binding proteins and are characterized by a helix-turn-helix motif followed by a β hairpin with the loop connecting the strands of the β hairpin constituting the wing, “W.” The Mbp1 ortholog from *Magnaporthe oryzae* binds to DNA in a manner typical of winged helix domains (Clark et al., 1993), with the second helix of the helix-turn-helix motif inserting into the major groove of DNA and residues in the wing interacting with the minor groove (Figure 3C) (Liu et al., 2015). In the INI1 domain the first two helices correspond to the helix-turn-helix motif, with strands 3 and 4 being equivalent to the hairpin. The INI1, SnoN/DACH1/Ski, BCOR, and DNTTIP1 domains differ from most winged helix domains by having an additional helix in the loop that forms the wing.

### Conserved Residues Map to a Putative Nucleic Acid Binding Site

**Figure 2:**
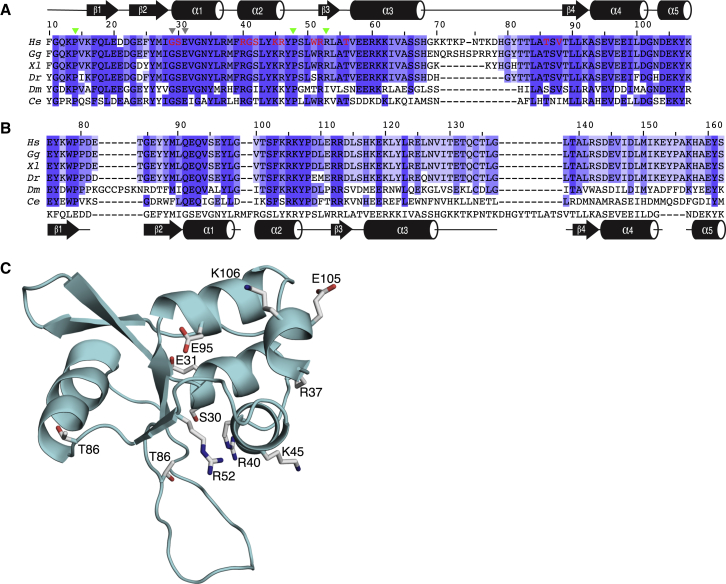
Sequence Conservation in the INI1 and BAF45a Domains (A) Sequence alignment of the N-terminal winged helix domains of INI1 homologs from representative species. The secondary structure of the domain is shown above the sequences, with sheets and helices indicated by arrows and cylinders. Species abbreviations: *Hs*, *Homo sapiens*; *Gg*, *Gallus gallus*; *Xl*, *Xenopus laevis*; *Dr*, *Danio rerio*; *Ce*, *Caenorhabditis elegans*. The numbering is for the human protein. Residues mutated in familial schwannomatosis are indicated by green triangles. Residues mutated in sporadic disease are indicated with gray triangles. Residues that undergo large changes in chemical shift upon DNA binding are colored red. (B) Structure-based alignment of the homologous domain in BAF45a proteins from the same species. The sequence and secondary structure of the INI1 domain are shown below the alignment. (C) Cartoon representation of the INI1 N-terminal domain, with the side chains of highly conserved residues shown as sticks.

A structure-based alignment of the N-terminal domains of INI1 homologs from a range of metazoan species is shown in Figure 2A. Many residues are invariant in proteins from humans to *Caenorhabditis elegans*. Some of these are buried hydrophobic residues, and others are glycines or proline residues in turns that also appear to be conserved for structural reasons. Several of the conserved residues, however, are solvent exposed and are likely to be functionally important. Most of these are in regions of the protein that interact with DNA in other winged helix domains (Figure 2C). These include Arg40 and Lys45 in the helix that in the Mbp1 DNA complex structure inserts into the major groove of DNA, Arg37 in the loop that precedes it, and Arg52 in the hairpin that follows it. Two residues, Ser30 and Glu31 in η1, which also contact DNA in the complex structure, are also completely conserved. Residues Thr56 and Thr88, which are at the beginning and end, respectively, of the loop that acts as the wing, are also highly conserved and only begin to be substituted with serine in some invertebrate INI1 homologs (Figure 2A). A structure-based alignment of the sequences of the BAF45a domain from the same species is shown in Figure 2B. Insertions and deletions are restricted to loops at the periphery of the fold, and many of the residues that make up the hydrophobic cores of the INI1 domain are conserved or replaced with other hydrophobic residues, suggesting that that the structure of the BAF45a domain is very similar to that of the INI1 domain. Several of the solvent-exposed residues conserved in INI1 are also conserved in the BAF45a domain, especially in helix 2 and in the first strand of the hairpin, suggesting that it may have a similar function.

### The INI1 N-Terminal Domain Binds dsDNA

**Figure 3:**
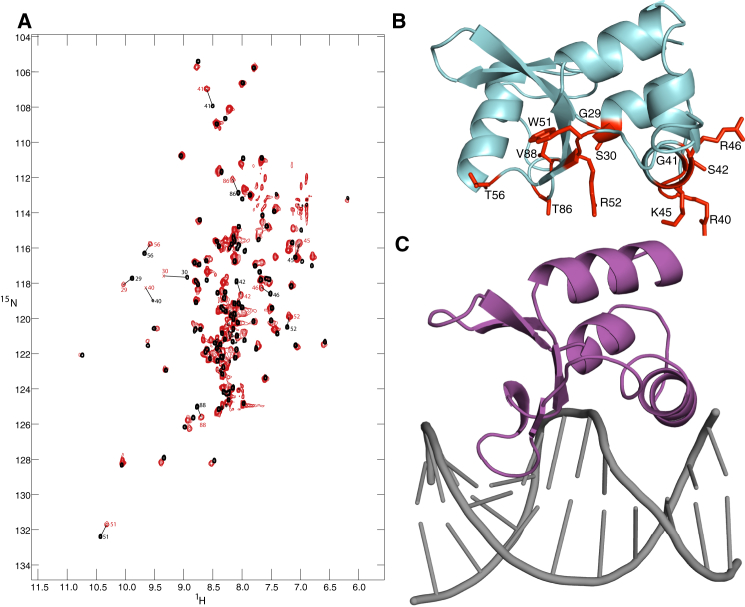
Binding of the INI1 Domain to dsDNA (A) ^1^H,^15^N-BEST-TROSY spectra of the INI1 domain free (black) and bound to a dsDNA oligonucleotide (red). Residues that undergo particular large changes in chemical shift upon DNA binding are indicated. (B) Cartoon representation of the INI1 N-terminal domain, with the side chains of residues that undergo large changes in chemical shift shown as sticks and colored red. (C) Cartoon representation of PCG2, the *Magnaporthe oryzae* ortholog of Mbp1, bound to its target DNA sequence.

The finding that many conserved residues map to the site used by other winged helix domains to interact with DNA suggested that the INI1 domain might have a similar function. As the sequence preference, if any, of the INI1 domain has not been established, we decided to test whether it could interact with a generic double-stranded (ds)DNA sequence by recording 2D BEST-TROSY spectra of ^15^N-labeled protein in the presence and absence of a palindromic DNA oligonucleotide. Addition of the oligonucleotide produces large changes in the chemical shift of a subset of residues (Figure 3A). Analysis of the dependence of the changes in chemical shift on the amount of oligonucleotide added shows that the bound and free peaks are in fast exchange on the NMR timescale, and gives an estimate of the dissociation constant in the high micromolar range which is similar to that seen for non-sequence-specific DNA interaction of proteins with this fold. A number of residues undergo especially large changes in chemical shift, and these are mapped onto a cartoon representation of the fold of the domain in Figure 3B. These residues include several in the helix predicted to insert into the major groove of the DNA based on the Mbp1 complex structure, as well as residues in the hairpin and wing regions that are expected to bind to the minor groove. Large changes in chemical shift are also observed for residues in the first helix, which is predicted to interact with the phosphate backbone of the DNA. These data suggest that the INI1 domain binds to dsDNA in a manner similar to that seen for other winged helix domains.

### Disease-Causing Mutations Either Disrupt the Fold of the Domain or Locate to the DNA Binding Site

**Figure 4:**
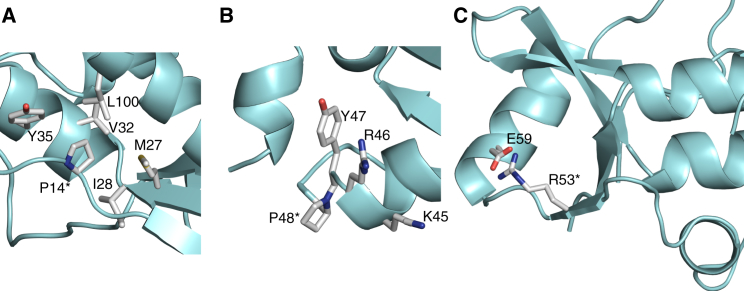
Residues Mutated in Familial Schwannomatosis Close-up views of the INI1 N-terminal domain showing the positions of Pro14 (A), Pro48 (B) and Arg53 (C). The mutated residues are indicated with an asterisk.

Germline mutations in the gene coding for INI1 are found in approximately 50% of inherited cases of schwannomatosis (Smith et al., 2014). The most common missense mutation is P14H (Boyd et al., 2008; Hadfield et al., 2008). Pro14 is in a region of extended strand that precedes the first β strand. The side chain of this residue, which is highly conserved, interacts with the side chains of hydrophobic residues in helices 1 and 4 (Figure 4A). Replacement of proline with a histidine at this position will disrupt these interactions and is expected to destabilize the domain. The missense mutations P48L and R53L have also been identified in familial schwannomatosis (Boyd et al., 2008; Christiaans et al., 2011). These residues, which are also highly conserved, are within the DNA binding site. Pro48 is in a turn at the end of helix 2 (Figure 4B). Replacement of this residue with a leucine would be expected to alter the conformation of this part of the protein. Arg53 is in the first strand of the β hairpin that forms the wing (Figure 4C). This residue is on the surface of the protein close to residues that change chemical shift upon addition of DNA, suggesting that it could be functionally important. The side chain of Arg53 interacts with Glu59 in helix 3 and thus may also contribute to the stability of the fold. Attempts to express the Arg53Leu mutant unsuccessfully suggested that the loss of this interaction destabilizes the fold of the domain. A mutation that changes amino acid 31 from a glutamate to a valine has also been identified in patients with familial schwannomatosis; however, it appears that the pathogenic impact of this mutation results from an alteration in mRNA splicing (Bacci et al., 2010). Glu31 is in helix 1 and is also predicted to be involved in ligand binding, and it is likely that this substitution would also be deleterious to the function of the domain. An in-frame deletion that removes residues 29 and 30 has been linked to familial schwannomatosis (Smith et al., 2014). These residues are at the N terminus of helix 1, and their deletion would be expected to be highly destabilizing.

Missense mutations have also been identified in sporadic schwannomatosis; it is not clear whether a particular substitution is pathogenic (Smith et al., 2012b). One of these mutations also affects Pro14, in this case replacing it with a serine. This substitution should also be expected to destabilize the protein, and it is clear that this mutation causes disease. A mutation that changes Gly29 in helix 1 to an arginine has also been associated with sporadic disease. This is predicted to be close to the DNA binding site, and the substitution of glycine for arginine at this position is likely to be functionally deleterious.

## Discussion

The appearance of a variant winged helix domain in both INI1 and BAF45a is presumably the result of the need for more intricate regulation of the SWI/SNF complex as it began to direct complex gene expression programs during the development of multi-cellular animals. The finding that structurally related domains are also present in a number of other metazoan chromatin-associated proteins suggests that domains with this fold may have a conserved role related to some aspect of chromatin biology. The data presented here argue strongly that the INI1 domain has a function involving binding to DNA. A sequence-specific DNA binding role appears to be unlikely, as the SWI/SNF complex is thought to be directed to specific sites within the genome by interacting with transcription factors. It is more probable that, as has been proposed for the homologous domain from DNTTIP1 (Itoh et al., 2015), it acts as a chromatin binding module, perhaps only binding with high affinity to DNA within a particular chromatin environment. Crosslinking studies of yeast SWI/SNF nucleosome complexes have shown that the INI1 homolog SNF5, which does not contain the winged helix domain, associates with the histone octamer and not with nucleosomal DNA (Dechassa et al., 2008). Assuming that the overall architecture of the complex is conserved in metazoa, the INI1 winged helix domain could be positioned to interact with DNA either in the bound nucleosome or in adjacent sequences. These interactions might be required for optimal remodeling activity or for the complex to act at particular regulatory elements. Perhaps it is the loss of these types of function that leads to disease. The data reported here provide a starting point for further efforts to establish the role played by this domain both in the SWI/SNF complex and other chromatin-associated proteins, and how mutations that disrupt its function cause disease.

## Experimental Procedures

The DNA encoding the first 115 residues of human INI1 was amplified from human cDNA by PCR and cloned into a modified pRSETA (Invitrogen) expression vector that produces proteins fused to the N-terminally His_6_-tagged lipoyl domain of *Bacillus stearothermophilus* dihydrolipoamide acetyltransferase. The resulting plasmids were transformed into *E. coli* C41 (DE3) cells. Cells were grown in 2XTY media at 37°C to mid-log phase and induced with 1 mM isopropyl β-D-1-thiogalactopyranoside. The temperature was reduced to 22°C and the cells were grown for a further 16 hr. Isotopically labeled domains were prepared by growing cells in K-MOPS minimal media containing ^15^NH_4_Cl and/or [^13^C]glucose. Cells where lysed by sonication, and the fusion protein was purified by Ni^2+^-NTA affinity chromatography and then dialyzed overnight in the presence of TEV protease, which cleaves the fusion protein after the lipoyl domain. A second Ni^2+^-NTA affinity chromatography step was carried out to remove the lipoyl domain, and the IN11 domain was further purified by gel filtration using a HiLoad 26/60 Superdex 75 column (GE Healthcare). The elution volume of the domain was consistent with it being monomeric.

### NMR Measurements

NMR measurements were made using either a Bruker DRX800 or DRX600 spectrometer equipped with triple-resonance cryoprobes at 25°C. NMR samples were typically 1.5 mM in 90% H_2_O, 10% D_2_O, containing 20 mM potassium phosphate (pH 6.5), 100 mM NaCl, and 5 mM β-mercaptoethanol. Assignments were obtained using standard NMR methods. Heteronuclear NOE spectra were obtained using the standard Bruker pulse program.

### Structure Determination

Distance restraints were obtained from the analysis of 2D and 3D NOE spectra. Backbone phi/psi dihedral angle restraints were obtained from chemical shifts using TALOS+ (Shen et al., 2009). The three-dimensional structures of the INI1 domain were calculated using the standard torsion angle dynamics-simulated annealing protocol in the program CNS 1.2 (Brunger, 2007). Structures were accepted where no distance violation was >0.25 Å and no dihedral angle violation >5°. Figures were made with the program PyMOL (Schrödinger). The coordinates and the NMR restraints have been deposited in the PDB with the codes PDB: 5aj1 and r5aj1mr, respectively.

### DNA Binding Experiments

Chemical shift perturbation analysis was used to characterize the interaction of the INI1 winged helix domain and dsDNA. A ^15^N-labeled INI1 winged helix domain sample was titrated with unlabeled 24-bp dsDNA. A palindromic oligonucleotide 5′-GGAATTGTGAGCGCTCACAATTCC-3′ was chemically synthesized (Integrated DNA Technologies), heated to 98°C for 10 min, and annealed at room temperature to form dsDNA. ^1^H,^15^N-BEST-TROSY spectra of the INI1 domain were collected at each point of the titration to monitor changes in ^1^H and ^15^N resonance frequencies of the domain induced by dsDNA binding.

## Figures and Tables

**Table 1:** Summary of Conformational Constraints and Statistics for the 20 Accepted NMR Structures of the INI1 N-Terminal Domain

**Structural Constraints**	

Intra-residue	963
Sequential	549
Medium-range (2 ≤ |*i* − *j*| ≤ 4)	361
Long-range (|*i* − *j*| > 4)	576
Dihedral angle constraints	30
TALOS constraints	162
Distance constraints for 42 hydrogen bonds	84
Total	2,725

**Statistics for Accepted Structures**	

Statistical parameters (±SD)	
Rmsd for distance constraints (Å)	0.0083 ± 0.0007
Rmsd for dihedral constraints (°)	0.471 ± 0.031
Mean CNS energy term (±SD) (kcal mol^−1^)	
E (overall)	117.26 ± 8.49
E (van der Waals)	34.13 ± 2.96
E (distance constraints)	13.26 ± 2.32
E (dihedral and TALOS constraints)	5.20 ± 0.68
Rmsd from the ideal geometry (±SD)	
Bond lengths (Å)	0.0014 ± 0.0001
Bond angles (°)	0.322 ± 0.0080
Improper angles (°)	0.235 ± 0.013
Average atomic rmsd from the mean structure (±SD) (Å)	
Residues 717–785 (N, Cα, C atoms)	0.376 ± 0.139
Residues 717–785 (all heavy atoms)	0.874 ± 0.121

rmsd, root-mean-square deviation.

## Supplemental Information

Document S1. Figure S1

Document S2. Article plus Supplemental Information

## References

[bib1] Altschul S.F., Madden T.L., Schaffer A.A., Zhang J., Zhang Z., Miller W., Lipman D.J. (1997). Gapped BLAST and PSI-BLAST: a new generation of protein database search programs. Nucleic Acids Res..

[bib2] Bacci C., Sestini R., Provenzano A., Paganini I., Mancini I., Porfirio B., Vivarelli R., Genuardi M., Papi L. (2010). Schwannomatosis associated with multiple meningiomas due to a familial SMARCB1 mutation. Neurogenetics.

[bib3] Boyd C., Smith M.J., Kluwe L., Balogh A., Maccollin M., Plotkin S.R. (2008). Alterations in the SMARCB1 (INI1) tumor suppressor gene in familial schwannomatosis. Clin. Genet..

[bib4] Brunger A.T. (2007). Version 1.2 of the crystallography and NMR system. Nat. Protoc..

[bib5] Cheng S.W., Davies K.P., Yung E., Beltran R.J., Yu J., Kalpana G.V. (1999). c-MYC interacts with INI1/hSNF5 and requires the SWI/SNF complex for transactivation function. Nat. Genet..

[bib6] Christiaans I., Kenter S.B., Brink H.C., van Os T.A., Baas F., van den Munckhof P., Kidd A.M., Hulsebos T.J. (2011). Germline SMARCB1 mutation and somatic NF2 mutations in familial multiple meningiomas. J. Med. Genet..

[bib7] Clark K.L., Halay E.D., Lai E., Burley S.K. (1993). Co-crystal structure of the HNF-3/fork head DNA-recognition motif resembles histone H5. Nature.

[bib8] Cole C., Barber J.D., Barton G.J. (2008). The Jpred 3 secondary structure prediction server. Nucleic Acids Res..

[bib9] Dechassa M.L., Zhang B., Horowitz-Scherer R., Persinger J., Woodcock C.L., Peterson C.L., Bartholomew B. (2008). Architecture of the SWI/SNF-nucleosome complex. Mol. Cell. Biol..

[bib10] Euskirchen G., Auerbach R.K., Snyder M. (2012). SWI/SNF chromatin-remodeling factors: multiscale analyses and diverse functions. J. Biol. Chem..

[bib11] Hadfield K.D., Newman W.G., Bowers N.L., Wallace A., Bolger C., Colley A., McCann E., Trump D., Prescott T., Evans D.G. (2008). Molecular characterisation of SMARCB1 and NF2 in familial and sporadic schwannomatosis. J. Med. Genet..

[bib12] Holm L., Rosenstrom P. (2010). Dali server: conservation mapping in 3D. Nucleic Acids Res..

[bib13] Hulsebos T.J., Plomp A.S., Wolterman R.A., Robanus-Maandag E.C., Baas F., Wesseling P. (2007). Germline mutation of INI1/SMARCB1 in familial schwannomatosis. Am. J. Hum. Genet..

[bib14] Itoh T., Fairall L., Muskett F.W., Milano C.P., Watson P.J., Arnaudo N., Saleh A., Millard C.J., El-Mezgueldi M., Martino F. (2015). Structural and functional characterization of a cell cycle associated HDAC1/2 complex reveals the structural basis for complex assembly and nucleosome targeting. Nucleic Acids Res..

[bib15] Jagani Z., Mora-Blanco E.L., Sansam C.G., McKenna E.S., Wilson B., Chen D., Klekota J., Tamayo P., Nguyen P.T., Tolstorukov M. (2010). Loss of the tumor suppressor Snf5 leads to aberrant activation of the Hedgehog-Gli pathway. Nat. Med..

[bib16] Junco S.E., Wang R., Gaipa J.C., Taylor A.B., Schirf V., Gearhart M.D., Bardwell V.J., Demeler B., Hart P.J., Kim C.A. (2013). Structure of the polycomb group protein PCGF1 in complex with BCOR reveals basis for binding selectivity of PCGF homologs. Structure.

[bib17] Kim S.S., Zhang R.G., Braunstein S.E., Joachimiak A., Cvekl A., Hegde R.S. (2002). Structure of the retinal determination protein Dachshund reveals a DNA binding motif. Structure.

[bib18] Lessard J., Wu J.I., Ranish J.A., Wan M., Winslow M.M., Staahl B.T., Wu H., Aebersold R., Graef I.A., Crabtree G.R. (2007). An essential switch in subunit composition of a chromatin remodeling complex during neural development. Neuron.

[bib19] Liu J., Huang J., Zhao Y., Liu H., Wang D., Yang J., Zhao W., Taylor I.A., Peng Y.L. (2015). Structural basis of DNA recognition by PCG2 reveals a novel DNA binding mode for winged helix-turn-helix domains. Nucleic Acids Res..

[bib20] Masliah-Planchon J., Bieche I., Guinebretiere J.M., Bourdeaut F., Delattre O. (2015). SWI/SNF chromatin remodeling and human malignancies. Annu. Rev. Pathol..

[bib21] Narlikar G.J., Sundaramoorthy R., Owen-Hughes T. (2013). Mechanisms and functions of ATP-dependent chromatin-remodeling enzymes. Cell.

[bib22] Nyman T., Tresaugues L., Welin M., Lehtio L., Flodin S., Persson C., Johansson I., Hammarstrom M., Nordlund P. (2010). The crystal structure of the Dachshund domain of human SnoN reveals flexibility in the putative protein interaction surface. PLoS One.

[bib23] Shen Y., Delaglio F., Cornilescu G., Bax A. (2009). TALOS+: a hybrid method for predicting protein backbone torsion angles from NMR chemical shifts. J. Biomol. NMR.

[bib24] Shidlovskii Y.V., Krasnov A.N., Nikolenko J.V., Lebedeva L.A., Kopantseva M., Ermolaeva M.A., Ilyin Y.V., Nabirochkina E.N., Georgiev P.G., Georgieva S.G. (2005). A novel multidomain transcription coactivator SAYP can also repress transcription in heterochromatin. EMBO J..

[bib25] Smith M.J., Walker J.A., Shen Y., Stemmer-Rachamimov A., Gusella J.F., Plotkin S.R. (2012). Expression of SMARCB1 (INI1) mutations in familial schwannomatosis. Hum. Mol. Genet..

[bib26] Smith M.J., Wallace A.J., Bowers N.L., Rustad C.F., Woods C.G., Leschziner G.D., Ferner R.E., Evans D.G. (2012). Frequency of SMARCB1 mutations in familial and sporadic schwannomatosis. Neurogenetics.

[bib27] Smith M.J., Wallace A.J., Bowers N.L., Eaton H., Evans D.G. (2014). SMARCB1 mutations in schwannomatosis and genotype correlations with rhabdoid tumors. Cancer Genet..

[bib28] Taylor I.A., Treiber M.K., Olivi L., Smerdon S.J. (1997). The X-ray structure of the DNA-binding domain from the *Saccharomyces cerevisiae* cell-cycle transcription factor Mbp1 at 2.1 A resolution. J. Mol. Biol..

[bib29] Tolstorukov M.Y., Sansam C.G., Lu P., Koellhoffer E.C., Helming K.C., Alver B.H., Tillman E.J., Evans J.A., Wilson B.G., Park P.J. (2013). Swi/Snf chromatin remodeling/tumor suppressor complex establishes nucleosome occupancy at target promoters. Proc. Natl. Acad. Sci. USA.

[bib30] Versteege I., Sevenet N., Lange J., Rousseau-Merck M.F., Ambros P., Handgretinger R., Aurias A., Delattre O. (1998). Truncating mutations of hSNF5/INI1 in aggressive paediatric cancer. Nature.

[bib31] Wilson J.J., Malakhova M., Zhang R., Joachimiak A., Hegde R.S. (2004). Crystal structure of the dachshund homology domain of human SKI. Structure.

[bib32] Xu R.M., Koch C., Liu Y., Horton J.R., Knapp D., Nasmyth K., Cheng X. (1997). Crystal structure of the DNA-binding domain of Mbp1, a transcription factor important in cell-cycle control of DNA synthesis. Structure.

