# Supplementary material for: The SWI/SNF Subunit INI1 Contains an N-Terminal Winged Helix DNA Binding Domain that Is a Target for Mutations in Schwannomatosis
Source: Structure. 2015 Jul 7;23(7):1344–9. doi: 10.1016/j.str.2015.04.021 (PMC4509781; doi:10.1016/j.str.2015.04.021)
Supplement: Document S1. Figure S1 [file mmc1.pdf]

Structure, Volume *23*

Supplemental Information

The SWI/SNF Subunit INI1 Contains an N-Terminal Winged Helix DNA Binding Domain that Is a Target for Mutations in Schwannomatosis

Mark D. Allen, Stefan M.V. Freund, Giovanna Zinzalla, and Mark Bycroft

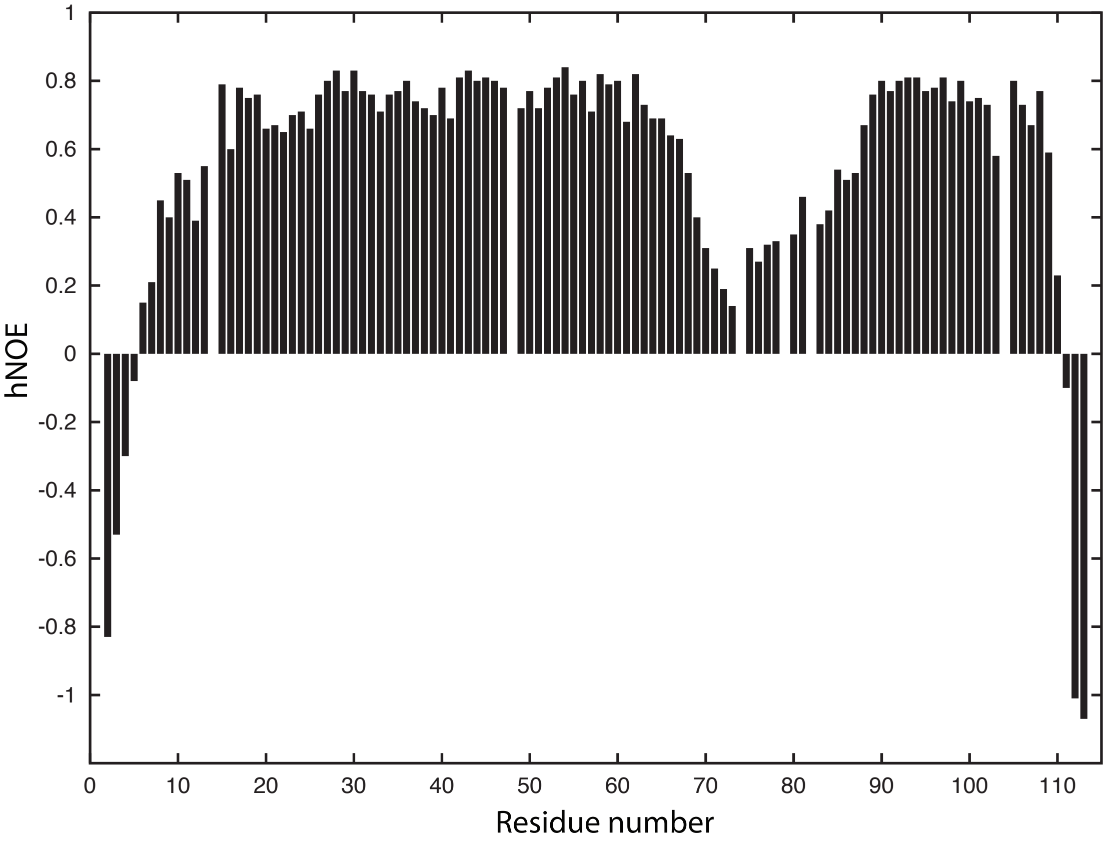

1H–15N heteronuclear NOE values for the INI1 N-terminal domain
